# Atypical Regressive Corneal Endothelial Cysts in Long-Term Confocal Follow-Up: A Case Report

**DOI:** 10.1097/MD.0000000000000564

**Published:** 2015-03-06

**Authors:** Adrian Smedowski, Edward Wylegala, Lukasz Wojcik, Dorota Tarnawska

**Affiliations:** From the Clinical Department of Ophthalmology (AS, EW, LW, DT), Faculty of Medicine and Division of Dentistry in Zabrze, Medical University of Silesia, Panewnicka; Department of Physiology (AS), Medical University of Silesia, Medykow; and Department of Biophysics and Molecular Physics (DT), Institute of Physics, University of Silesia, Uniwersytecka, Katowice, Poland.

## Abstract

Corneal endothelium is formed of 1 layer of mitochondria-rich cubic cells whose main role is to maintain corneal transparency. Corneal endothelial disorders represent group of both inherited and noninherited and may affect proper vision.

A 36-year-old male patient with suspicion of corneal endothelial dystrophy underwent visual acuity, intraocular pressure, the basic slit-lamp examination, anterior segment optical coherence tomography (AS-OCT) (Visante, Carl Zeiss Meditec, Dublin, CA), and corneal confocal microscopy in vivo (Rostock Cornea Module, Heidelberg Engineering Retina Tomograph III, Heidelberg, Germany). During the 3-year observation the patient reported symptoms mainly in the right eye. Slit-lamp examination revealed endothelial changes, much more pronounced in the right eye. Examination by the AS-OCT Visante showed hyperreflective dots within the right corneal endothelium. In order to assess endothelial cell morphology, analysis using corneal confocal microscopy in vivo was performed. Scans revealed presence of single endothelial deposits and severe cell changes of different morphology in both eyes. In the right eye, less pronounced changes of the polymorphic structure—polygonal guttas in different stages, linear and branched loss with “nuclear-like” formations and accompanying sediments. In the left eye, severe homomorphous polygonal “guttas-like” changes with “nuclear-like” formations were observed. Endothelial cysts’ features were dynamically changing during follow-up time with different effects on the patient's clinical state.

Corneal confocal microscopy allows accurate imaging of the endothelial cells and their detailed characteristics. Structural changes within the endothelial cells are not always proportional to visual acuity and slit-lamp image. The presented case is an example of an unusual corneal endothelial syndrome with probably nondystrophic background due to observed dynamic state with regressive tendency.

## INTRODUCTION

The term “dystrophy” is commonly used to describe an inherited disorder, fulfilling certain criteria. In ophthalmology, the term “corneal dystrophy” has no strictly defined borders. It is a group of corneal disorders usually with the following features: inherited, noninflammatory, typically bilateral, symmetric, slowly progressive (regression in dystrophy development is unusual), and without relationship to environmental or systemic factors.^1^ However, in some cases corneal dystrophies can coexist with other systemic disabilities (macular dystrophy or amyloidosis, which is often called lattice dystrophy type II, Schnyder dystrophy) or can develop unilateral (posterior polymorphous endothelial dystrophy). On the other hand, there are some corneal abnormalities which are excluded from the corneal dystrophies group, despite fulfilling defined conditions (such as “cornea plana”—inherited, bilateral, usually not related to systemic abnormalities).^2^ Corneal endothelial dystrophies concern diseases characterized by corneal endothelial cells layer abnormalities, what usually leads to slowly progressive degeneration of corneal endothelium, decreasing of cells density and affecting visual acuity.^3^

On the contrary, there are nondystrophic endothelial syndromes that include variants of iridocorneal endothelial syndromes (ICE) and endothelial (preendothelial) deposits. In such cases, changes might be observed unilaterally with tendency for both progression and regression while they are secondary pronunciation of other eye disorders (inflammatory changes, iris and iridocorneal angle pathologies, iatrogenic repercussion).^4,5^

The aim of this report is to describe abnormal phenotype of corneal endothelium in a 36-year-old patient, with features of clinical regression accompanied by progressive endothelial pathology.

## CASE PRESENTATION

The study was approved by the institutional review board of the Medical University of Silesia, Katowice, Poland, and informed consent was obtained from the patient after providing an explanation of the nature and possible consequences of the study.

A 36-year-old patient with suspicion of corneal endothelial dystrophy underwent the following examination: best-corrected visual acuity (BCVA), intraocular pressure (IOP), refraction, the basic slit-lamp examination including anterior and posterior segment examination before and after mydriasis, corneal imaging with anterior segment optical coherence tomography (AS-OCT) including central corneal thickness (CCT) and corneal topography measurements (Visante, Carl Zeiss Meditec, Dublin, CA), and corneal confocal microscopy in vivo (Rostock Cornea Module, Heidelberg Engineering Retina Tomograph III, Heidelberg, Germany). During the 3-year follow-up, the patient reported symptoms of decreased and blurred vision mainly in the right eye. In anamnesis, no history of any type of ocular surgery (including refractive surgery), no family history of ocular diseases, no use of contact lenses or eye drops usage at present or in the past. In blood samples, no inflammatory markers elevation was observed (C-reactive protein level, erythrocyte sedimentation rate). The patient was referred to our outpatient clinic after he underwent detailed ophthalmic examination in a foreign clinic (the Netherlands), where corneal changes have been described as a case of an “unknown dystrophy.”

**FIGURE 1:**
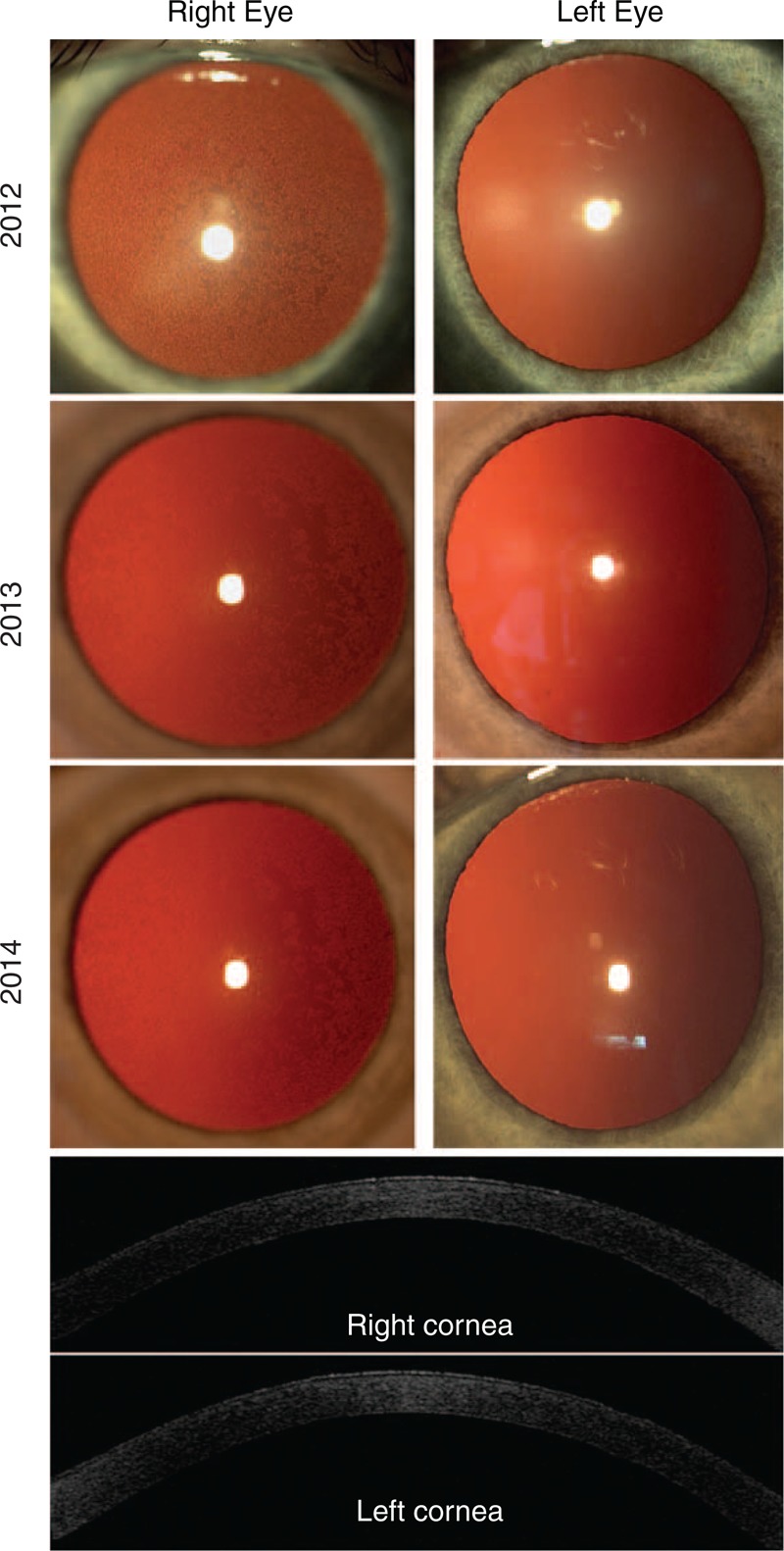
Slit-lamp photographs of anterior segment after mydriasis. Posteriorly localized changes of alveolar and linear morphology, much more pronounced in the right eye. During follow-up time visible regrouping of endothelial changes that became eccentrically localized. AS-OCT Visante revealed hyperreflective dots within endothelium of the right cornea and discreet hyperdensity of corneal stroma in both eyes.

During the 3-year follow-up the clinical status of the patient changed. Decimal BCVA over the years of observation ranged from 20/63 to 20/25 for the right eye and was constant at 20/20 for the left eye (Table 1). IOP values were within normal limits (below normative 21 mm Hg). Slit-lamp examination revealed posteriorly localized changes of alveolar and linear morphology, much more pronounced in the right eye. No other abnormalities were described within the anterior and posterior segments of both eyes, including gonioscopy and corneal topography results (Figure 1). Mean CCT was 499 ± 15 μm in the right and 500 ± 19 μm in the left eye; corneal pachymetric maps showed no abnormalities. Examination with the AS-OCT Visante showed hyperreflexive dots and discreet hyperdensity of corneal stroma mostly within the right cornea (Figure 1).

**TABLE 1:**
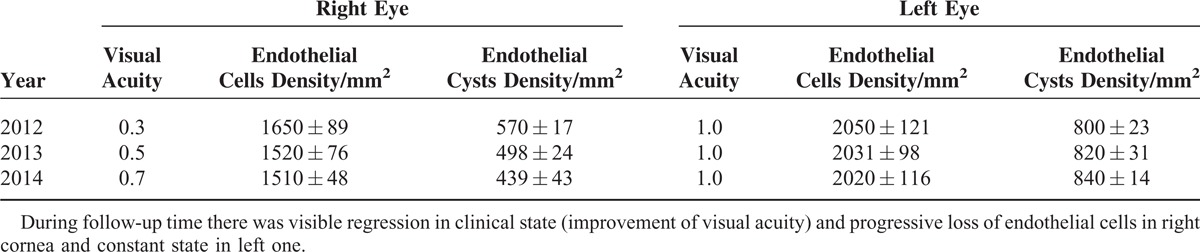
Visual Acuity and Endothelial Layer Measurements Including Density of Endothelial Cells and Endothelial Cysts

**FIGURE 2:**
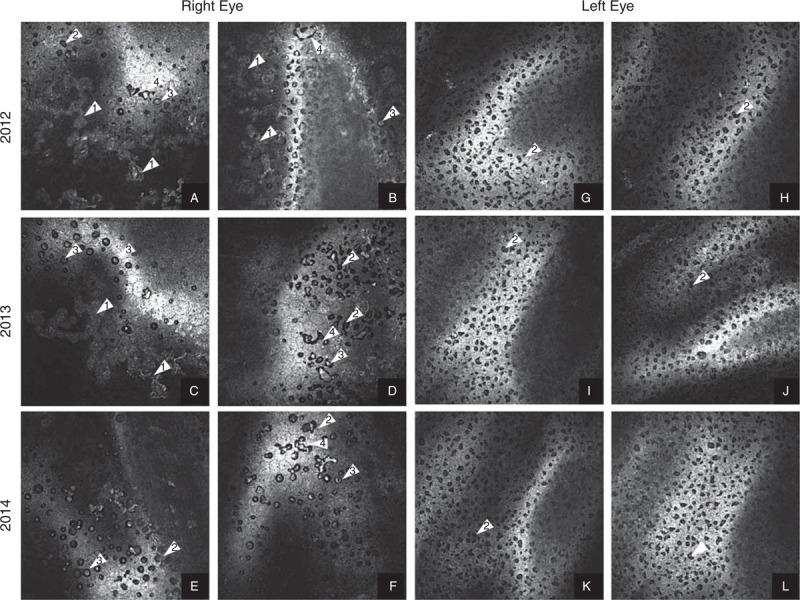
A–L. Corneal confocal microscopy in vivo. Over the years 2012 to 2014 in right cornea reduction of sediments (1) and presence of polygonal, polymorphous guttas in different stage (2) with “nuclear-like” creations (3) and linear and branched dropouts (4) were observed. In left cornea–dominated polygonal, homomorphous guttas in different stage (2).

In order to assess endothelial cell morphology, analysis using corneal confocal microscopy in vivo was performed. The scans revealed presence of massive endothelial deposits in the right eye and severe cell changes of different morphology in both eyes. In the right eye, we found less pronounced changes of the polymorphic structure—polygonal guttas in different stages, linear and branched loss with “nuclear-like” formations, and cells polymegathism (Figure 2A–L). In the left eye, more severe monomorphous polygonal “guttas-like” changes with small “nuclear-like” formations, with no signs of polymegathism, were visible (Figure 2 A-L). Endothelial cells density and density of endothelial cysts, determined using “cell count” module of corneal confocal microscopy, are presented in Table 1.

Between regular examination visits, we observed dynamic regression in clinical status, which was concurrent with BCVA improvement. Endothelial changes in the right eye presented regressive features for their localization (clearing of central corneal area) as well as for decreasing presence of sediments; however, progressive endothelial cells loss was observed. Regression was accompanied by releasing of endothelial deposits material from endothelium to anterior chamber causing aqueous humor convection (probably noninflammatory). Both lenses showed no visible features of pseudoexfoliation (PEX) syndrome. In the left eye, endothelial abnormalities and clinical status remained stable.

## DISCUSSION

In the described case, suspected corneal endothelial dystrophy presents some features that could be characteristics for posterior corneal dystrophies, however, not fully matching any of already described dystrophies. There is also no clear relation to any nondystrophic secondary endothelial pathology. Changes appeared bilaterally, but they have asymmetric morphology. Severity of changes did not correlate with clinical state and (based on anamnesis) seemed not to be inherited.

In the case of posterior polymorphous corneal dystrophy (PPCD), changes appear mostly bilaterally and have similar morphology, however, can also be asymmetric. Clinical state usually does not correlate with severity of changes. In endothelial cells, dominant features are polymegathism, vesicular, band, and diffuse lesions. Additionally, peripheral iridocorneal adhesions and elevated IOP can appear.^6^ In Fuchs endothelial corneal dystrophy (FECD) characteristics are symmetric guttas of different size and round shape, polymegathism and pleomorphism of cells, with presence of corneal edema due to degeneration of the corneal endothelium. Changes involve usually both eyes.^1^ In congenital hereditary endothelial dystrophies (CHEDs), changes are bilateral, symmetric or asymmetric. Massive endothelial cells degeneration cause corneal edema from the time of birth or shortly after. In cell structure, the characteristic features are atrophy, vacuolization, and metaplasia with keratotic epithelial cells.^7,8^ X-linked endothelial corneal dystrophy (XECD)—with characteristic “moon crater–like” endothelial changes—develop in early age.^9^ Based on description of endothelial dystrophies, there might be similarities observed between changes in the patient's right eye and PPCD; however, the expression is not typical. The left eye does not present any features of already known dystrophies. In this case, very likely explanation is a coexistence of 2 different types of corneal dystrophies or 2 different phenotypes of the same dystrophy. Descriptions of this kind of rare combinations can be found in the literature.^10–12^ Considering 3-year follow-up of presented patient's disease, it is rather unlikely to search for dystrophic background, mostly due to regression in patient's clinical state that is untypical for dystrophic intercourse. In comparison with available confocal description of ICE syndrome, where authors describe presence of epithelioid cells within endothelium, hyperreflectivity of cells nuclei, sac-like blisters, and ruptured blisters, revealed no such cell abnormalities in the presented report.^4,13,14^

## CONCLUSION

Corneal confocal microscopy allows accurate imaging of the endothelial cells and their detailed characteristics. Structural changes within the endothelial cells are not always proportional to visual acuity and slit-lamp image. The presented case is an example of an unusual corneal endothelial phenotype with probably nondystrophic background due to observed dynamics of endothelial changes with regressive tendency.
